# Toxicological Profile of the Pain-Relieving Antioxidant Compound Thioctic Acid in Its Racemic and Enantiomeric Forms

**DOI:** 10.3390/antiox9080749

**Published:** 2020-08-14

**Authors:** Elena Lucarini, Elena Trallori, Daniele Tomassoni, Francesco Amenta, Carla Ghelardini, Alessandra Pacini, Lorenzo Di Cesare Mannelli

**Affiliations:** 1 Department of Neuroscience, Psychology, Drug Research and Child Health (NEUROFARBA)-Pharmacology and Toxicology Section, University of Florence, Viale Gaetano Pieraccini, 6, 50139 Florence, Italy; elena.lucarini@unifi.it (E.L.); elena.trallori@stud.unifi.it (E.T.); carla.ghelardini@unifi.it (C.G.); 2 School of Biosciences and Veterinary Medicine, University of Camerino, Via Gentile III Da Varano, 62032 Camerino, Italy; daniele.tomassoni@unicam.it; 3 Section of Human Anatomy, School of Pharmacy, University of Camerino, Via Madonna delle Carceri 9, 62032 Camerino, Italy; francesco.amenta@unicam.it; 4 Department of Experimental and Clinical Medicine, Anatomy and Histology Section, University of Florence, Largo Brambilla 3, 50134 Florence, Italy; alessandra.pacini@unifi.it

**Keywords:** neuropathic pain, thioctic acid, antioxidant, food supplement, caspase-3

## Abstract

Thioctic acid is a multipotent antioxidant compound existing as dextrorotatory (+), eutomer and naturally occurring and levorotatory (−). It has been proven to help fight many pathologies and is sold as racemate. In agreement with studies claiming a greater biopotency of the eutomer compared to the levorotatory compound, we recently preclinically and clinically showed that (+) thioctic acid is a pain-reliever as effective as double-dosed racemate. We investigated acute and subchronical toxicity of (+/−) thioctic acid, (−) thioctic acid, (+) thioctic acid and (+) salt thioctic acid on Sprague–Dawley rats. For acute toxicity, compounds were administered intraperitoneally (i.p.) with a single-injection at 125, 240, 360, 480 µmol/kg, then rodents were tested for motorial coordination and minimum lethal dose (LDmin). A subtoxic dose (360 µmol/kg) was administered i.p. for 15 days and we finally evaluated motorial impairment, glycemia, organ toxicity, and apoptosis state. Acutely administered, the highest doses of all thioctic acid compounds negatively affected motorial ability and (−) thioctic acid LDmin resulted higher than the others. Subchronic administrations caused overall body weight loss, motorial impairment, mass loss in some organs. (+/−) and (−) thioctic acid injections enhanced caspase-3 activity in some organs, (−) enantiomer-treated animals displayed more marked organ toxicity signs. Together with our previous study on the biologic role of enantiomers, these data suggest a therapeutic use of (+) enantiomer-based formulations, thus lowering dose and toxicity without affecting the positive effects brought by the drug.

## 1. Introduction

**Figure 1:**
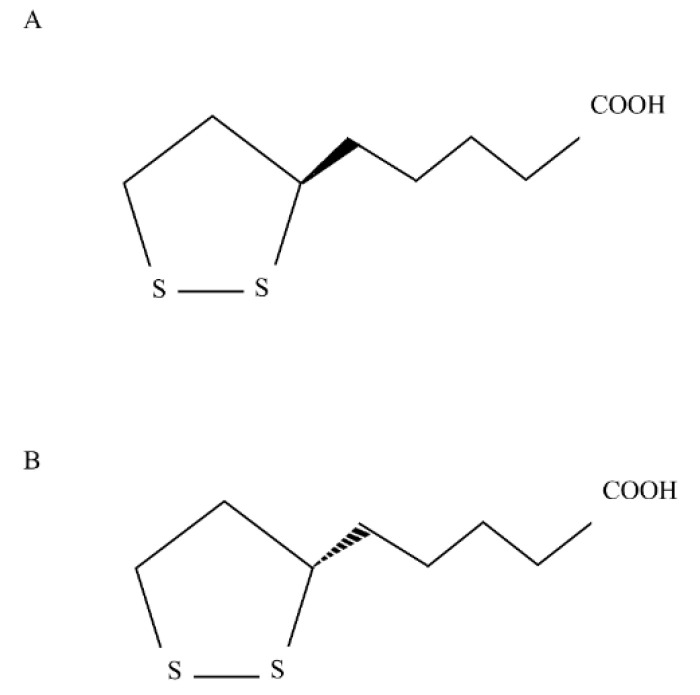
Chemical structure of (**A**) (+) thioctic acid and (**B**) (−) thioctic acid.

Thioctic acid (chemical formula: C_8_H_14_O_2_S_2_) is a dithiol eight-carbon molecule [1] with a chiral center which induces its isomerization into two optical enantiomers: (+) thioctic acid, the eutomer and (−) thioctic acid. The production of both two isomers occurs during non-enantioselective chemical (nonbiologic) synthesis (Figure 1) [2]. The eutomer is synthetized de novo in mammalian mitochondria by the enzyme lipoic acid synthase (LASY), from octanoic acid and cysteine [3]. Only (+) thioctic acid is synthetized by the cells and is biologically active: it is part of a mitochondrial complex which contributes to the synthesis and degradation of glycine. In addition—thanks to its amide linkage to lysine residue—(+) thioctic acid acts as a cofactor for some critical mitochondrial enzymes: pyruvate dehydrogenase (PDH), branched chain α-keto-acid dehydrogenase (KDH), α-ketoglutarate dehydrogenase (KGDH) [4]. Thioctic acid has a strong antioxidant activity explicated through different mechanisms exerted by the redox pair thioctic acid/dihydrothioctic acid: scavenging free radicals, restoring endogenous antioxidant molecules (such as vitamins A, C, E and glutathione) and chelating metal ions, thus reducing the production of reactive oxygen species [4,5]. Therefore, it has become therapeutically attractive for fighting oxidative stress underlying many disease processes such as polyneuropathies, diabetes complications, hepatopathies, and hypertension [6,7,8,9]. Since dietary sources (liver, heart, kidney, broccoli, spinach, etc.) contain low quantities of thioctic acid resulting in scarce bioavailability [5,10], this acid is sold as drug or dietary supplement in racemic form that increased its bioavailability [4]. Many studies highlighted that the eutomer has more biopotency than (−) thioctic acid [11,12,13] and, moreover, (+) enantiomer was proved to be longer available in the plasma than (−) enantiomer [14]. Previous studies of our groups fitted perfectly in this context: in a model of peripheral neuropathy induced by chronic constriction injury of sciatic nerve in hypertensive rats, we showed the analgesic, antioxidant and neuroprotective efficacy of a chronic administration of alpha lipoic acid, being (+) thioctic acid more active than (−) enantiomer and as active as double dose of racemate or even more than it [12,15].

The present study attempted to outline an acute and subchronic toxicological profile of thioctic acid enantiomers and racemic form. To assess acute toxicity profile, we evaluated motorial impairment and defined a minimum lethal dose (LDmin); under a prolonged treatment (15 days) at a sublethal dose, we investigated motorial impairment, hematic values and then ex vivo analysis ensued to outline subchronic toxicity profile of the substances. In summary, our data reported the presence of some toxic effects following acute and subchronic treatments with all compounds, but (+) enantiomer displayed a slightly lower subchronic toxicological profile, with no differences between acid and salt form, than (−) enantiomer and racemic form. The little toxicity and higher biologic activity of the dextrorotary enantiomer suggest the therapeutic use of the eutomer alone, thus allowing reduction of dosage and general toxicity.

## 2. Materials and Methods

### 2.1. Animals

The present study was carried on male Sprague–Dawley rats (Envigo, Varese, Italy), which weighed approximately 200–250 g at the beginning of the experimental procedure. Rats were accommodated in a laboratory animal facility (CeSAL, Centro Stabulazione Animali da Laboratorio, University of Florence) and used one week after their arrival. Each cage (size 26 × 41 cm^2^) housed four rats, which were given a standard laboratory diet and tap water ad libitum. Animals were kept at 23 ± 1 °C with a 12 h light/dark cycle, starting light at 7 a.m. All animal manipulations were carried out according to the directive 2010/63/EU of the European Parliament and of the European Union council (22 September 2010, amended by Regulation (EU) 2019/1010) on the protection of animals used for scientific purposes. The ethical policy of the University of Florence complies with the guide for the care and use of laboratory animals of the US National Institutes of Health (NIH Publication No. 85–23, revised 1996; University of Florence assurance number: A5278-01). The below described experiments were approved by the Italian Ministry of Health (No. 54/2014-B) and by the animal subjects review board of the University of Florence. Since the study involved animals, the experiments were reported according to ARRIVE guidelines [16]. All efforts were made to reduce at minimum extent animal suffering and the number of animals used.

### 2.2. Animal Treatments

Thioctic acid, as racemate, acid and tromethamine salt (+) enantiomer, (−) enantiomer, was pursued from Sintactica (Milan, Italy). Compounds were solubilized in NaOH-supplemented physiologic solution and buffered to 7.4 pH by adding HCl. In acute toxicity tests, rats were divided in groups (*n* = 6 per group) and treated with intraperitoneal (i.p.) injection of 125, 240, 360, 480 µmol/kg of each compound (corresponding to 25, 50, 75 and 100 mg/kg of (+/−) thioctic acid) while controls group (*n* = 6 per group) received the same amounts of vehicle. Acute toxicity tests were performed 30 min and 2 h following injections. Subchronic toxicity tests were performed 24 h after the last injection of 15-day treatments with 360 µmol/kg of each thioctic acid isoform.

### 2.3. Rotarod Test

Motor coordination was measured by the rotarod test according to [17]. The apparatus (Ugo Basile, Varese, Italy) consisted of a base platform and a rotating rod with a diameter of 3 cm and a non-slippery surface. The rod was placed at a height of 15 cm from the base. The rod—30 cm in length—was divided into 5 equal sections by 6 disks. Thus, up to five mice were tested simultaneously on the apparatus, with a rod rotating speed of 10 revolutions per minute. The integrity of motor coordination was assessed on the basis of the number of falls from the rod and the overall time of continuous walking in 10 min (cutoff time 600 s). A training pretest session was performed 24 h before acute treatment and at the beginning of the subchronic treatment.

### 2.4. Collection of Blood and Glycemia Analysis

Blood was collected immediately after sacrifice into Eppendorf tubes containing heparin (20 μL, 25,000 IU/5 mL). Plasmatic fraction was separated by spinning at 2000× *g* for 15 min and glucose levels were measured using Elite apparatus (Bayer, Leverkusen, Germany).

### 2.5. Tissue Explant

Following subchronic toxicity tests, rats were sacrificed by cervical dislocation and tissues explant was performed: thymus, lung, liver, spleen, kidney, suprarenal glands, skeletal muscle *digitorum longus* and spinal cord were collected. A portion of them was immediately frozen with liquid nitrogen for enzymatic and immunoblotting experiments; the rest was fixed in situ with 10% formalin in phosphate buffered saline (pH 7.4) and then bathed in sucrose gradients.

### 2.6. Caspase-3 Activity

A 20-mg portion of tissue was homogenized in lysis buffer containing Tris HCl 200 mM pH 7.5, NaCl 2 M, EDTA 20 mM, Triton X-100 0.2% and centrifuged for 5 min at 5000× *g*. Fifty microliters of the supernatants were incubated with 25-μM fluorogenic peptide caspases 3 substrate rhodamine 110 bis-(N-CBZ-L-aspartyl-L-glutamyl-L-va-lyl-L-aspartic acid amide) (EnzChek^®^ Caspase-3 Assay Kit, Molecular Probes, Milano, Italy) at 25 °C for 30 min in a buffer solution composed by PIPES 10 mM pH 7.4, EDTA 10 mM, CHAPS 0.5%, modifying experimental protocol from [18]. The amount of cleaved substrate per sample was measured in a 96-well plate fluorescent spectrometer (PerkinElmer, Milan, Italy) setting the following wavelengths: excitation at 496 nm and emission at 520 nm.

### 2.7. Histological Analysis

The liver and kidney were removed, cut into small pieces and fixed in 10% neutral buffered formalin. After dehydration in gradual ethanol (50% to 100%), they were cleared in xylene, paraffin-embedded and cut in consecutive 5-µm-thick sections. Sections were stained with hematoxylin and eosin dye and Azan–Mallory and observed under a light microscope (objective Zeiss Plan Apo 10, Oberkochen, Germany) at an original magnification of 20× and 40×.

### 2.8. Statistical Analysis

Analysis were performed on 6 rats for each treatment. All assessments were made by researchers blinded to animal treatments. Results were expressed as means ± SEM and the analysis of variance was performed by one-way ANOVA. A Fisher’s significant difference procedure was used as post hoc comparison. *p*-values less than 0.05 were considered significant. Data were analyzed using the “Origin” software version 9 (OriginLab, Northampton, MA, USA).

## 3. Results

### 3.1. Acute Toxicity

**Figure 2:**
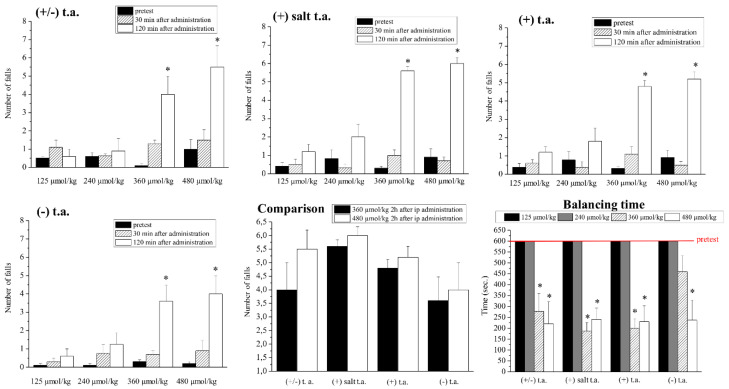
Effects of acute toxicity treatments: ataxia (Rota Rod test). The graphs represent: the number of falls observed 30 and 120 min after treatments with increasing concentrations (125, 240, 360 and 480 µmol/kg) of thioctic acid (t.a.) isoforms (racemate, (+) tromethamine salt enantiomer, (+) acid enantiomer, (−) enantiomer); a comparison among all groups of treatments at 360 and 480 µmol/kg two hours after the injection; the time of balanced walking after 2 h. Each value represents the mean ± SEM of 6 rats per group. * *p* < 0.05 vs. pretest.

The present study focused on acute and subchronic toxicity induced by thioctic acid isoforms. At first, we studied acute toxicity: we evaluated ataxia by rotarod test as shown by the results in Figure 2. The rodents were injected i.p. with 125-, 240-, 360-, 480-µmol/kg thioctic acid isoforms and after 30 and 120 min we reported the falls from the rotating rod and the balance time on the rod. Thirty minutes after the administration, independently from any dose and isoform used, thioctic acid did not alter the number of falls recorded during control animals’ performances (not shown) which resemble pretest results. On the contrary, all the isoforms at 360 and 480 µmol/kg induced a statistically significant increase of falls from the rotating rod two hours after the injections: racemic form and both (+) enantiomers (acid and tromethamine salt compounds) caused from five-fold to six-fold rise, while the effect of (−) enantiomer was slightly minor. The comparison panel clearly showed no significant changes in number of falls at the two highest doses (360 and 480 µmol/kg) among the different groups of treatment. Time of balance on the rotating rod was expressed in seconds: 600 s were arbitrarily chosen as time period useful to highlight motor impairments and were set as cutoff time. This time was reached by all rodents during pretest performed the day before. The data concerning time of balance revealed a similar toxicity pattern (“balancing time”): at 125 and 240 µmol/kg, all thioctic acid isoform allowed the rodents to walk continuously on the rod for 600 s, like the pretest, while at 360 and 480 µmol/kg the racemic mixture and the (+) enantiomers reduced by 70% this time of walking; the same reduction was observed when administering 480 µmol/kg of (−) enantiomer.

Another investigated parameter was the minimum lethal dose (LDmin): racemic mixture, acid and salt (+) enantiomers displayed lethality at 960 µmol/kg, while (−) isoform was scaled up till 1450 µmol/kg, before being mortal (Table 1).

### 3.2. Subchronic Toxicity

#### 3.2.1. Body Weight and Hematic Parameters

**Figure 3:**
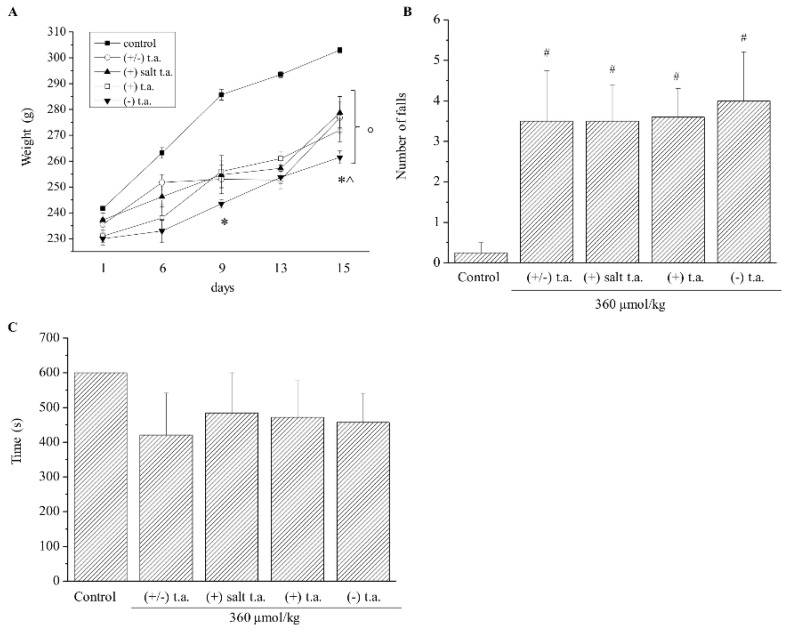
Subchronic toxicity treatments: body weight and ataxia. Effects of 15-day subchronic toxicity treatment with 360 µmol/kg of thioctic acid (t.a.) racemate, (+) enantiomer tromethamine salt, (+) enantiomer acid, (−) enantiomer on (**A**) body weight, (**B**) number of falls during 10 min of walking on the rotating rod and (**C**) time of continuous walking on the rotating rod. Each value represents the mean ± SEM of 6 rats per group. ° *p* < 0.05 vs. animals treated with physiological solution from Day 6, * *p* < 0.05 vs. animals treated with racemate, ^ *p* < 0.05 vs. animals treated with (+) enantiomers, # *p* < 0.05 vs. animals treated with physiological solution.

**Figure A1:**
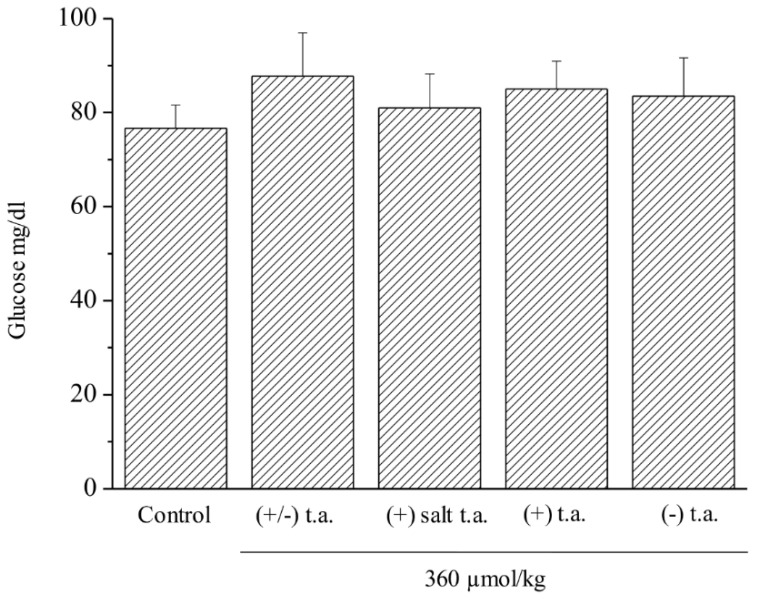
Subchronic toxicity treatments: glycaemia levels. Effects of 15-day subchronic toxicity treatments with 360 µmol/kg (+/−) t.a., (+) salt t.a., (+) t.a., (−) t.a. on plasmatic levels of glucose.

Considering these preliminary data, we moved on studying subchronic toxicity effects: for two weeks treatments were repeated administering every day i.p. all the substances at the minimum toxic dose 360 µmol/kg, which was outlined by previously performed acute toxicity experiments. The first effect to be evaluated was the variation of body weight, by measuring it every day, from Day 0 to Day 15 (Figure 3A). After six days of injections with all isoforms of thioctic acid, rodents lost weight in comparison to control animals treated with physiological solution, which gained weight. If treatments with (+) salt and acid enantiomers induced anorexic effects to the same extent, a noticeable major impact on body weight was exerted by (−) enantiomer administrations: a significant difference of outcomes was registered on Day 9 and 15 than racemic mixture and on Day 15 than (+) isoform injections. We then evaluated again motor coordination ability: the number of falls increased by about 4-fold, independently from the isoform injected (Figure 3B), whereas the walking time period on the rotating rod was not affected by the substances (Figure 3C). Thioctic acid injections did not modify glycemia values since at the end of the treatments they measured around 80 mg/dL in all groups (Appendix A, Figure A1).

#### 3.2.2. Ex vivo Analysis: Organ Mass

**Figure 4:**
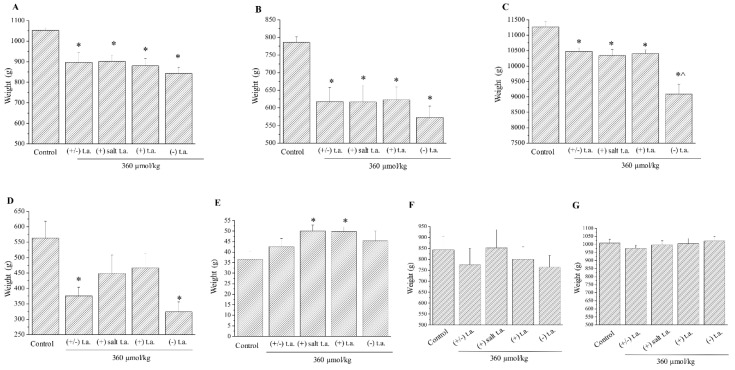
Subchronic toxicity treatments: organ weight. Effect of 15-day subchronic toxicity treatment with 360-µmol/kg thioctic acid (t.a.) isoforms (racemate, (+) tromethamine salt enantiomer, (+) acid enantiomer, (−) enantiomer) on: (**A**) heart, (**B**) spleen, (**C**) liver, (**D**) thymus, (**E**) adrenal gland, (**F**) right lung and (**G**) kidney. Each value represents the mean ± SEM of 6 rats per group. * *p* < 0.05 vs. control; ^ *p* < 0.05 vs. racemate, (+) thioctic acid.

By the end of the treatment the animals were sacrificed and ex vivo analysis was carried on. First, main organs were weighed: in all treated groups heart (Figure 4A) and spleen (Figure 4B) lost 15% and 23% of mass than control, respectively; liver lost 7% of weight in almost all treated groups and this reduction rose up to 18% when administrating (−) enantiomer (Figure 4C); the mass of thymus was nearly halved only by injections of racemic and (−) enantiomeric thioctic acid, decreasing from 564 g to 376 g and 324 g, respectively (Figure 4D). We noticed that, when animals were treated with (+) enantiomers, suprarenal gland’s weight rose in a statistically significant way, increasing by around 1.5-fold from 36.7 to 50 g (Figure 4E), while all the treatments had no effects on lungs and kidneys (Figure 4F,G). A summary of organ mass variation is outlined in Appendix A, Table A1.

#### 3.2.3. Ex Vivo Analysis: Apoptotic State

**Figure 5:**
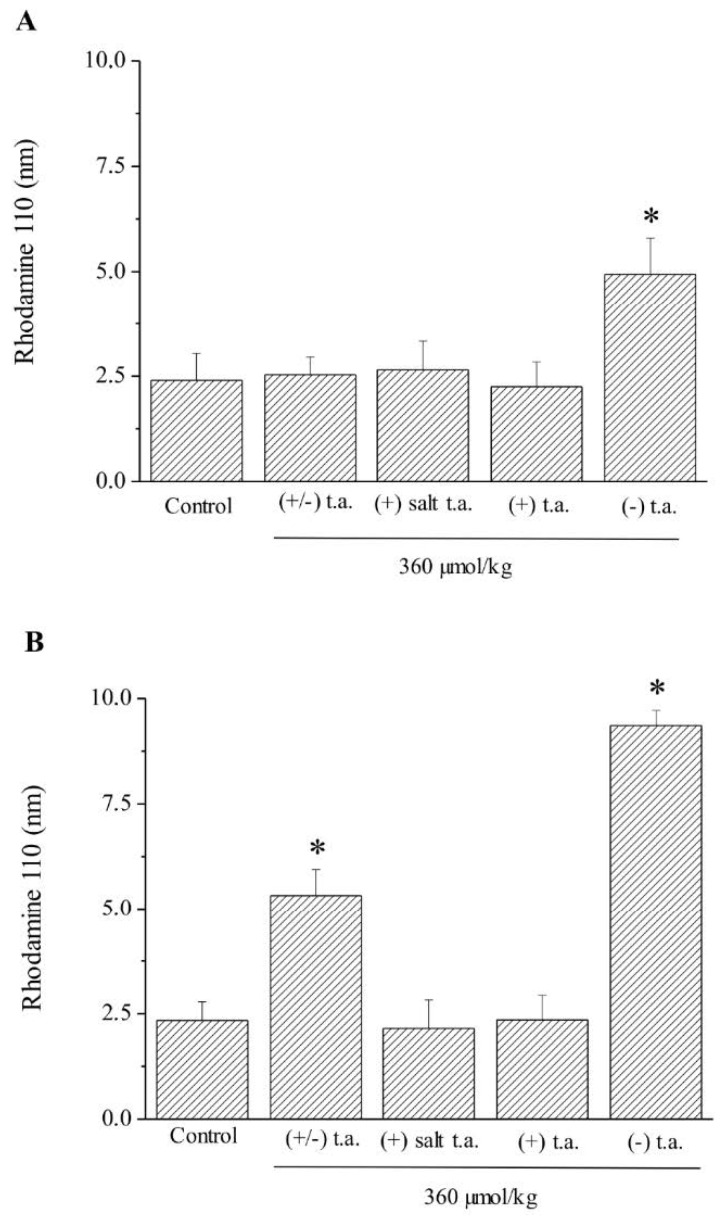
Subchronic toxicity treatments: caspase-3 activity in kidney and spinal cord. Effects of 15-day subchronic toxicity treatment with 360-µmol/kg thioctic acid (t.a.) isoforms ((+/−) racemate, (+) tromethamine salt enantiomer, (+) acid enantiomer, (−) enantiomer) on enzymatic activity of Caspase-3 in (**A**) kidney and (**B**) spinal cord. The amount of cleaved substrate rhodamine 110 was measured by setting as wavelengths: excitation at 496 nm and emission at 520 nm. Each value represents the mean ± SEM of 6 rats per group. * *p* < 0.05 vs. controls treated with physiological solution.

**Figure A2:**
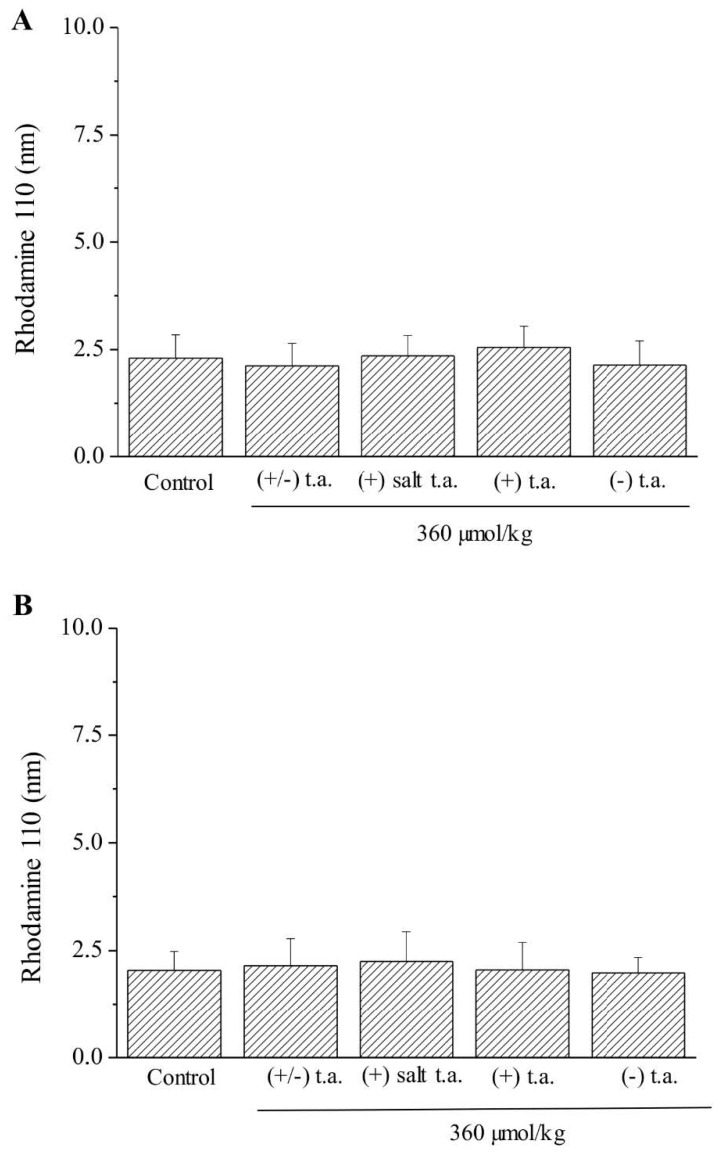
Subchronic toxicity treatments: caspase-3 activity in liver and skeletal muscle. Effects of 15-day subchronic toxicity treatments with 360 µmol/kg (+/−) t.a., (+) salt t.a., (+) t.a., (−) t.a. on enzymatic activity of Caspase-3 in liver (**A**) and skeletal muscle (**B**). The amount of cleaved rhodamine 110 substrate was measured by excitation at 496 nm and emission at 520 nm. Each value represents the mean ± SEM of 6 rats per group.

The apoptotic state was checked by the measurement of the catalytic activity of caspase-3, the key protein of apoptosis phenomenon, in order to observe cellular suffering in the kidney, the spinal cord (Figure 5A,B), the liver and the skeletal muscles (Appendix A, Figure A2) [19]. Enzymatic activity of caspase-3 was measured via the amount of cleaved rhodamine 110 substrate. Caspase-3 activity was doubled (Figure 5A) in kidney of animals treated with (−) enantiomer; it was 2 and 3.5-fold more than control in the spinal cord of racemic and (−) enantiomer treated rodents, respectively (Figure 5B). In the liver and in the skeletal tissue, treatments induced no alteration in enzymatic activity (Figure A2).

#### 3.2.4. Ex Vivo Analysis: Organ Toxicity

**Figure 6:**
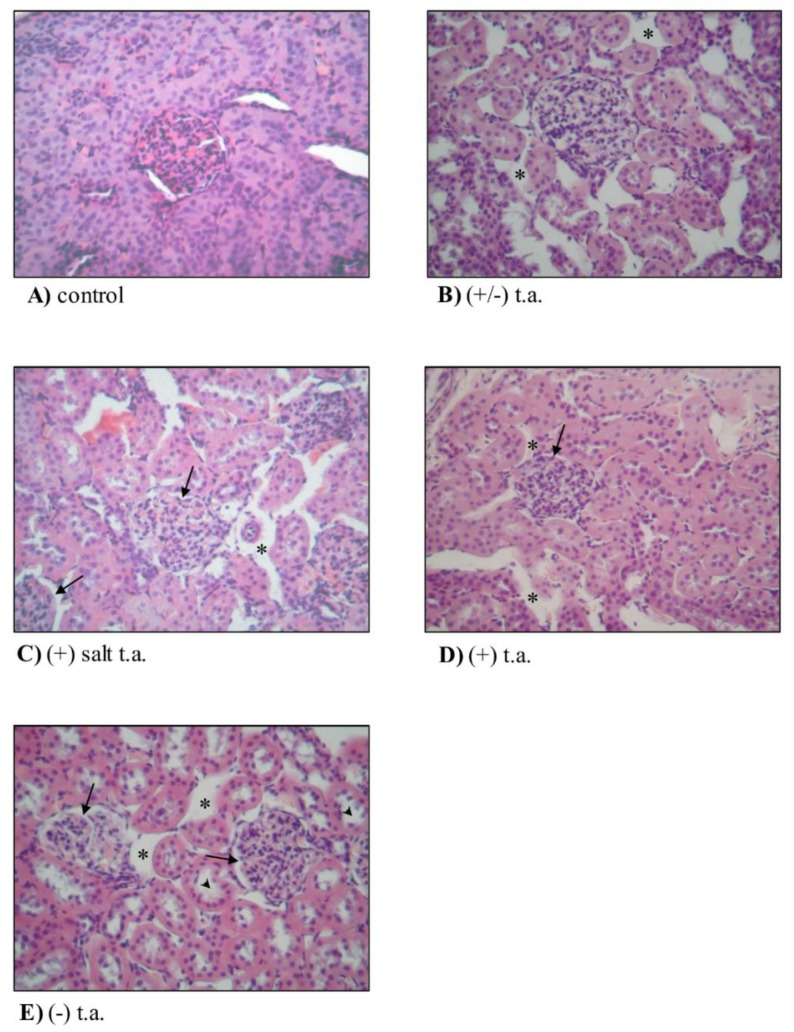
Subchronic toxicity treatments: kidney histology. Effects of 15-day subchronic toxicity treatments with 360-µmol/kg thioctic acid (t.a.) isoforms on histological features of kidney. Histological analysis was performed on kidney samples explanted from rodents treated with (**A**) physiological solution, (**B**) racemic thioctic acid, (**C**) (+) tromethamine salt enantiomer, (**D**) (+) acid enantiomer and (**E**) (−) enantiomer. Asterisks, arrows and arrowheads point to morphological changes. Staining with hematoxylin–eosin technique; original magnification 20×.

Finally, kidney and liver were investigated from a histological point of view, by processing 5 micrometers sections with hematoxylin–eosin staining and Azan–Mallory technique. In Figure 6A the biopsy preparation of control renal cortex displayed, even at low magnification, normal renal corpuscles, which were instead absent from renal medulla (image not shown). The images of kidney sections from rodents treated with thioctic acid compounds ((+/−) thioctic acid, (+) salt enantiomer, (+) acid enantiomer and (−) enantiomer) showed alterations: the renal interstitium was edematous (asterisks in Figure 6B–E) even if lacking in mononucleate cellular infiltrates such as macrophages and lymphocytes. Glomeruli were normal, but Bowman’s spaces were more dilated (arrows in Figure 6C–E). Because of substances’ toxicity, there was a pathologic increase of luminal diameter of renal tubules, which was evident in all treated group samples compared to control ones, but more remarkably in (−) isoform treated rats (arrowheads in Figure 6E).

**Figure 7:**
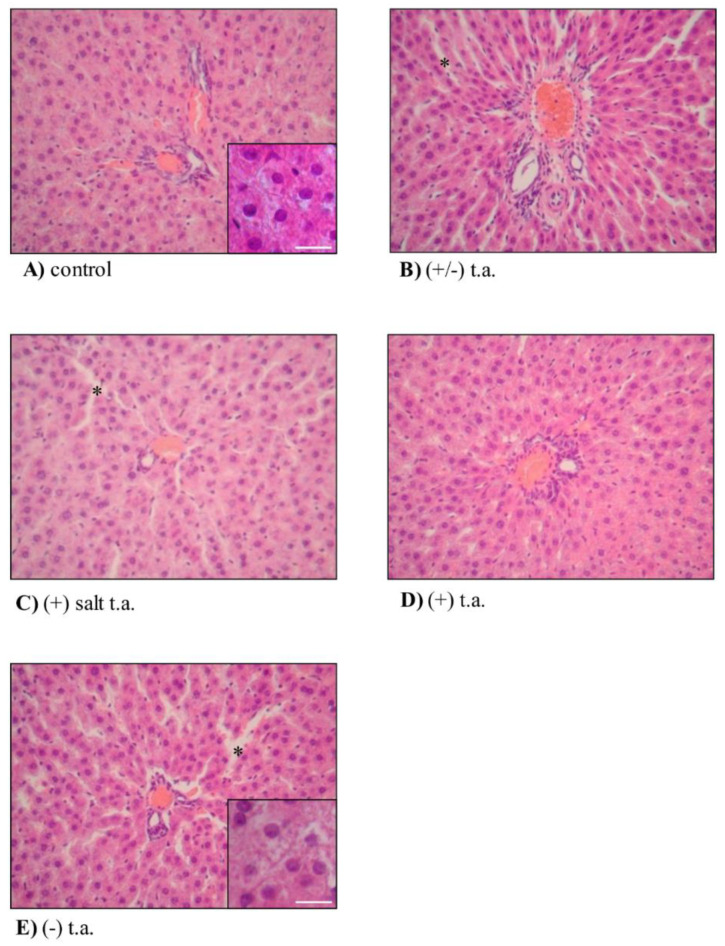
Subchronic toxicity treatments: liver histology. Effects of 15-day subchronic toxicity treatment with 360-µmol/kg thioctic acid isoforms on histological features of liver. Histological analysis was performed on liver samples explanted from rodents treated with (**A**) physiological solution, (**B**) racemic thioctic acid, (**C**) (+) tromethamine salt enantiomer, (**D**) (+) acid enantiomer and (**E**) (−) enantiomer. Asterisks point to morphological changes. Staining with hematoxylin–eosin techniques; original magnification 20×; insert scale bars = 200 µm.

**Figure A3:**
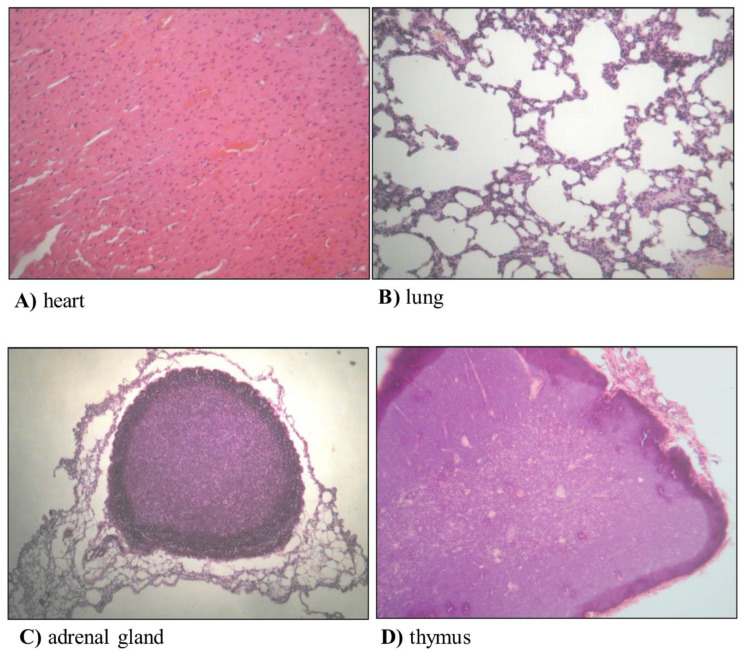
Subchronic toxicity by (−) oriented enantiomer treatment: organ histology. Effects of 15-day subchronic toxicity treatments with 360 µmol/kg (−) thioctic acid on histological features of heart (**A**), lung (**B**), adrenal gland (**C**), thymus (**D**). Staining with hematoxylin–eosin techniques; 20× magnification.

Turning attention to the liver, the hepatic parenchyma was not macroscopically affected by repeated treatments with thioctic acid, since we reported the presence of normal capsule made of dense connective tissue (images not shown). Microscopically (Figure 7), no alteration was observed in the hematic irroration, because portal spaces were visible and carried many vases with different diameters. First signs of toxicity effects were observed on hepatic lobules, characterized by irregular undefinable borders, probably due to an edematous state, but again with no cellular infiltrate (images not shown). At increased magnification (20×), treated hepatocytes (Figure 7B–E) were slightly different from their physiological shape (Figure 7A): they were not polyhedral, nor with round nuclei, but they were slightly swollen and displayed a lower staining affinity than that of controls (inserts in Figure 7A,E). On the other side, the scattered chromatin content, the peripheral nucleoli, the presence of binucleated cells followed the physiological pattern. Uncolored spots in the cytoplasm were probably due to technical procedures, such as glycogen and lipid extraction, therefore they did not point to morpho-functional alterations: as a proof, cytoplasm was strongly eosinophil because of the high number of mitochondria and, in addition, numerous free and rough endoplasmic reticulum-associated ribosomes provoked a fine basophil granularity in the background. Sinusoids appeared to be coated with thin endothelial cells, bearing dense and flattened nuclei and scarcely colored cytoplasm. Treatments with every isoform of thioctic acid caused sinusoids to lightly dilate (asterisks in Figure 7B,C,E), especially around the portal spaces, more prominently when using (−) enantiomer (Figure 7E). From the histological analysis of heart, lung, adrenal and suprarenal glands and thymus we did not observe any alteration which could be linked to thioctic acid treatments, both with racemic and enantiomeric forms; we only show sections of organs of (−) isoform treated animals in Appendix A, Figure A3.

## 4. Discussion

Thioctic acid is an organosulfur compound existing as two optical enantiomers: (+) and (−) thioctic acid. It has a strong antioxidant activity explicated by the redox couple thioctic acid/dihydrothioctic acid through different mechanisms: scavenging free radicals, restoring endogenous antioxidant molecules and chelating metal ions, thus reducing in turn the production of reactive oxygen species [4,5]. The (+) oriented eutomer was reported to be more biologically active than (−) enantiomer by a plethora of studies [11,12,13,20], also in agreement with our recent preclinical studies on the pain-alleviating effects of thioctic acid which concluded that the eutomer was more effective than the (−) enantiomer and as beneficial as the double-dose of racemic thioctic acid [12,15]. The present comparison study represents a novel point of view in the already existing literature context of preclinical and clinical toxicology of thioctic acid: we report signs of light toxicity induced from both racemate and enantiomers, with no relevant differences between the dextrorotary and levorotatory enantiomers except for some discrepancies in subchronic treatment. Following the currently accepted table of rodent to human dose conversion, the maximum dose of 100 mg/kg, used in this study, is comparable to 600 mg in humans [21,22], a commonly used clinical dosage [23].

Acute and subchronical neurotoxicity was evaluated through rotarod test, which assesses ataxia and motorial impairment, two signs of neurological damage [24]. In our study, acute intraperitoneal administrations of all forms of thioctic acid were toxic at 360 and 480 µmol/kg (corresponding to 75 mg/kg and 100 mg/kg of racemic form) because they similarly altered neurological and motorial capabilities of the rodents in all groups of treatment; (+/−) and (+) thioctic acid decreased time of walking at 360 and 480 µmol/kg, while (−) thioctic acid only at 480 µmol/kg. Interestingly, minimum lethal dose was lower for the racemic form and the eutomer (960 µmol/kg) than the threshold of (−) enantiomer (1450 µmol/kg), maybe because of minor biological efficacy of the latter. This result was not in line with a study from Cremer and colleagues who reported that the acute oral LD50 was in excess of 2000 mg/kg, the NOAEL in excess of 61.9 mg/kg per day and the LOAEL in excess of 121 mg/kg per day [25]. Many other studies, conducted on other animal species, reported different thresholds: an oral LD50 of 400–500 mg/kg was described for dogs by [6]; Hill and collaborators claimed a 10-fold or more sensitivity of cats than of rats, humans and dogs [26]. The discordance of minimum LD may be due to the route of administration: intraperitoneal injections are characterized by larger absorption area and more rapid absorption than oral administration; on the other hand the compound is subjected to hepatic first-pass elimination.

Subchronic treatments with sublethal dose (360 µmol/kg) lasted 15 days. We observed weight loss in all groups of treatment than control, but injections with levorotatory compound had a major anorexic effect over two days than racemate group and (+) salt group. This side effect may be result of an anorexic ability of the compounds, reported also by Cremer and coworkers, in a 2-year toxicity study on 181 mg/kg oral intake per day and in a 8-week study, led by Timmers and colleagues, on the effects of 0.5% racemic thioctic acid added to the high-fat and low-fat diet of rats [27,28]. There was no homogeneous trend in our absolute organ weight data: only the masses of heart and spleen were equally reduced by all types of administration, while levorotatory injections induced more pronounced consequences on liver and thymus masses. The racemic form lowered the weight of thymus as well; therefore weight loss could be ascribed to levorotatory action alone. On the other hand, a weight increase was noticed for adrenal glands of only (+) enantiomer groups. These weight alterations may be a compensatory response to an insult caused by thioctic acid.

All molecules also partially influenced motor coordination in a negative way at long term (two weeks treatment). Light disturbance of motorial ability was observed in a 4-week toxicity study by Cremer and his collaborators as well, which reported staggered and stilted gait in several female rats from 30 to 180 min after oral administration of 121 mg/kg racemic lipoic acid [25]. This low-level neurotoxicity must be balanced with the proved neuroprotective activity of the compound [12,15], attempting to understand the biologic activity of thioctic acid by all its different interactions. Hematic values assessment revealed no variation of glycemia at the end of the treatment, in agreement with the therapeutic effect of thioctic acid in sustaining and rising cellular glucose uptake [20].

We investigated the apoptotic state in the most relevant organs, through assessment of caspase-3 activity. Caspase-3 physiologically exists as an inactive pro-enzyme which is activated when apoptotic processes start, therefore its activation ensues and explains, a suffering state of the cell [19]. Our results cannot actually tell about an happening apoptotic state in any of the organs investigated, but there was a positive modulation of caspase-3 activity in kidney and in spinal cord from animals treated with (−) enantiomer and racemic form: prolonged injections with those isomers of thioctic acid activated caspase-3 pro-enzyme, creating a condition which could be read as more prone to trigger a pro-apoptotic cascade than a physiological one can be and suggesting an alarming cellular state. Being both administrations toxic, (−) enantiomer appeared to be the responsible for the results induced by racemic mixture. In literature we found voices opposing to our results: some studies reported an antiapoptotic activity of thioctic acid, when the dietary supplement was administered orally and at lower concentration (50 mg/kg) [29,30].

Histological analysis of kidney and liver revealed few signs of toxicity due to thioctic acid administrations, but levorotatory enantiomer produced more prominent effects than the other compounds. In the kidney, all the treatments with thioctic acid induced an edematous state of the renal interstitium with no infiltrating monocytes, which could be typical of acute inflammation response and Bowman’s capsule dilatation; lumen diameter appeared enlarged in all groups, but more markedly in levorotatory-treated group. Thioctic acid may disturb membrane homeostasis, leading to leakage and liquid accumulation—therefore ensuing slight edema formation and structure enlargement. A similar pattern emerged from the histological analysis of liver: the hepatic lobules had irregular borders and were larger, probably because of an edematous state lacking cellular infiltrate, maybe attributable to an acute inflammatory process; hepatocytes were swollen; sinusoids dilated, more remarkably in proximity of hepatic portals. Notably, sinusoidal dilatation was more evident in the group of animals treated with (−) enantiomer. All these data—gathered from histological analysis of kidney and liver—suggest slightly toxic effects produced by all isoforms of thioctic acid, but one of the side effects was more stressed when animals were treated for 15 days with (-) enantiomer. In the scientific literature, nephrotoxicity and hepatotoxicity were not confirmed by some studies reporting that thioctic acid fought and reduced renal and liver damage induced by other insults, probably because our subchronic dosage was higher (75 mg/kg vs. 50 and 25 mg/kg) and the route of administration was different (intraperitoneal vs. oral) [29,30,31].

## 5. Conclusions

The natural antioxidant compound thioctic acid—usually sold as racemic mixture—is considered health-improving in many pathologic states. Even if some clinical literature about the racemic formulation did not report side effects [9,32,33,34], other preclinical studies have revealed some signs of toxicity [25,27,28]. Our study highlighted little acute and subchronic toxicities of the compound in all forms, which, in our opinion, could be markedly reduced, exploiting the high-efficacy of the dextrorotatory eutomer: it could be administered alone and, as a consequence, the dose and the toxicity would be lowered.

## Figures and Tables

**Table 1:** Minimum lethal dose of i.p. injected (+/−) thioctic acid, (+) and (−) enantiomers.

Thioctic Acid Isoform	Minimum Lethal Dose (LDmin)
(+/−) thioctic acid	960 µmol/kg i.p.
(+) tromethamine salt thioctic acid	960 µmol/kg i.p.
(+) thioctic acid	960 µmol/kg i.p.
(−) thioctic acid	1450 µmol/kg i.p.

## Appendix A

**Table A1:** Organ mass variation following 15-day administration of (+/−) thioctic acid, (+) salt thioctic acid, (+) thioctic acid, (−) enantiomer.

Organ	Administration			
	(+/−) t.a.	(+) salt t.a.	(+) t.a.	(−) t.a.
Heart	−	−	−	−
Spleen	−	−	−	−
Liver	−	−	−	−*
Thymus	−	+/−	+/−	−
Right lung	+/−	+/−	+/−	+/−
Kidney	+/−	+/−	+/−	+/−
Adrenal glands	+/−	+	+	+/−

− negative variation, + positive variation, +/− no variation vs. untreated rats; + and − significantly variated vs. control; −* significantly variated vs. (+/−) t.a., (+) salt t.a., (+) t.a.
